# Beyond the Timeline: 1-Year Mortality Trends in Early Versus Late Prosthetic Valve Endocarditis

**DOI:** 10.1093/cid/ciae392

**Published:** 2024-07-27

**Authors:** Matthaios Papadimitriou-Olivgeris, Bruno Ledergerber, Berit Siedentop, Pierre Monney, Michelle Frank, Georgios Tzimas, Piergiorgio Tozzi, Matthias Kirsch, Jana Epprecht, Mathias van Hemelrijck, Omer Dzemali, Benoit Guery, Barbara Hasse

**Affiliations:** Infectious Diseases Service, Lausanne University Hospital and University of Lausanne, Lausanne, Switzerland; Infectious Diseases Service, Cantonal Hospital of Sion and Institut Central des Hôpitaux, Sion, Switzerland; Department of Infectious Diseases and Hospital Epidemiology, University Hospital Zurich and University of Zurich, Zurich, Switzerland; Department of Infectious Diseases and Hospital Epidemiology, University Hospital Zurich and University of Zurich, Zurich, Switzerland; Department of Cardiology, Lausanne University Hospital and University of Lausanne, Lausanne, Switzerland; Department of Cardiology, University Hospital Zurich and University of Zurich, Zurich, Switzerland; Department of Cardiology, Lausanne University Hospital and University of Lausanne, Lausanne, Switzerland; Department of Cardiac Surgery, Lausanne University Hospital and University of Lausanne, Lausanne, Switzerland; Department of Cardiac Surgery, Lausanne University Hospital and University of Lausanne, Lausanne, Switzerland; Department of Infectious Diseases and Hospital Epidemiology, University Hospital Zurich and University of Zurich, Zurich, Switzerland; Department of Cardiac Surgery, University Hospital Zurich and University of Zurich, Zurich, Switzerland; Department of Cardiac Surgery, University Hospital Zurich and University of Zurich, Zurich, Switzerland; Center for Translational and Experimental Cardiology, Department of Cardiology, University Hospital of Zurich and University of Zurich, Zurich, Switzerland; Infectious Diseases Service, Lausanne University Hospital and University of Lausanne, Lausanne, Switzerland; Department of Infectious Diseases and Hospital Epidemiology, University Hospital Zurich and University of Zurich, Zurich, Switzerland

**Keywords:** prosthetic valve endocarditis, embolic events, periannular complications, valve surgery, transcatheter aortic valve implantation (TAVI), infective endocarditis, heart failure

## Abstract

Among 302 episodes of prosthetic valve endocarditis (PVE), 1-year mortality was 31%. There was no evidence indicating that early-onset PVE within 6 months from valve surgery led to a worse outcome compared to late-onset PVE (21% vs 32%, *P* = .126), despite similar redo valve surgeries across both categories.

## Graphical Abstract

**Graphical Abstract:**
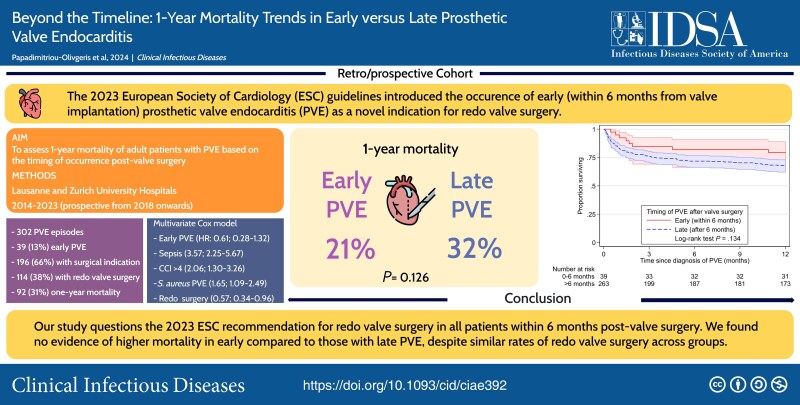
This graphical abstract is also available at Tidbit: https://tidbitapp.io/tidbits/beyond-the-timeline-one-year-mortality-trends-in-early-vs-late-prosthetic-valve-endocarditis

Prosthetic valve endocarditis (PVE) accounts for 20%–30% of all cases of infective endocarditis [1, 2]. The timing of PVE onset following valve surgery impacts microbiological characteristics and clinical presentations. Cases occurring within the initial 12 months are primarily attributable to direct contamination during surgery and/or hematogenous dissemination. Of note, during this time period, the prosthetic material remains devoid of endothelium, leaving the periannular space vulnerable to complications such as abscess formation, fistula development, and prosthesis dehiscence [1–5].

Owing to its heightened association with periannular invasion, valve surgery constitutes a crucial component in the management of PVE, with 40% of patients undergoing such intervention [1–7]. According to European Society of Cardiology (ESC) guidelines of 2015, updated in 2023, indications for valve surgery include heart failure, uncontrolled infection, and prevention of embolism [8, 9]. Notably, the 2023 edition of the ESC guidelines introduced the occurrence of PVE within 6 months following valve surgery as a novel indication for cardiac surgery, irrespective of other surgical indications [8]. In this context, our study aimed to assess the outcomes of patients with PVE by scrutinizing the timing of occurrence post–valve surgery.

## MATERIALS AND METHODS

We conducted this study at Lausanne University Hospital and University Hospital Zurich from January 2014 to June 2023 (retrospective inclusion from 2014 to 2017; prospective from 2018 onward). Approval was obtained from the ethics committees of the cantons of Vaud and Zurich (CER-VD-2017-02137, KEK-2014-0461; BASEC-2017-01140).

Inclusion criteria consisted of adults aged ≥18 years diagnosed with PVE. Prospective patients were required to provide written informed consent, whereas data usage was permitted in the absence of refusal for the retrospective cohort. We excluded subsequent PVE episodes occurring within 1 year of the initial episode. Data extracted from electronic health records included demographics, comorbidities, microbiological data, and cardiac surgery details.

The primary outcome was 1-year mortality post-PVE. We categorized our exposure of interest, the timing of PVE, into 2 groups: early PVE (≤6 months following valve surgery), and late PVE (>6 months). Valve surgery indications followed the 2023 ESC guidelines [8, 9], including heart failure, infection control, and prevention of embolism.

Statistical analyses involved χ^2^ or Fisher exact test for categorical variables and Mann–Whitney *U* test for continuous variables. We calculated the time from PVE onset until the first occurrence of 1 year, last visit, or death, whichever occurred first. Because of 6 censored observations, we assessed 1-year mortality using Kaplan-Meier techniques with the log-rank test. Subsequently, we applied uni- and bivariable Cox proportional hazards regression, stratified by hospital, to ascertain effect modifications and interactions with our exposure of interest. Given multiple PVE episodes in some patients, models were adjusted for clustering on individual patients. The final multivariable model included a priori sex and cardiac surgery, alongside non-collinear variables exhibiting significance in uni- and bivariable models. Age was included as part of the Charlson Comorbidity Index (CCI). In a sensitivity analysis, we excluded subsequent episodes of PVE. We conducted data analyses using Stata 18.0 SE (StataCorp, College Station, Texas).

## RESULTS

Among 1136 episodes of infective endocarditis, 302 (27%) PVE episodes occurred in 286 patients. Episodes were excluded due to the absence of PVE (818 [72%]) and subsequent PVE episodes occurring within 1 year from the initial episode (16 [1%]). Of the 302 episodes, 39 (13%) were classified as early PVE, while 263 (87%) were categorized as late PVE (Supplementary Table 1). Median age was 68 years (interquartile range [IQR], 49–76 years), and 58 individuals (20%) were female. One hundred thirty-seven (46%) had a CCI exceeding 4 points. *Staphylococcus aureus* was the prevailing pathogen (105 episodes [36%]). PVE was predominantly observed in the aortic valve (204 cases [68%]), with the majority involving biological valve (178 cases [60%]). One hundred eighteen episodes (40%) were associated with sepsis. Of the 203 episodes (67%) meeting the criteria for valve surgery as outlined in the 2023 ESC guidelines (excluding early PVE indication), 30 (15%) were attributed to heart failure, 185 (91%) to uncontrolled infection, and 54 (27%) to embolism prevention. The surgeons performed redo cardiac surgery in 114 (38%) episodes. However, 89 patients did not undergo surgery despite having an indication: 43 were deemed too high-risk due to advanced age and comorbidities, 35 did not require surgery according to cardiac surgeons, 7 were in a coma, and 4 refused the operation.

**Figure 1.**
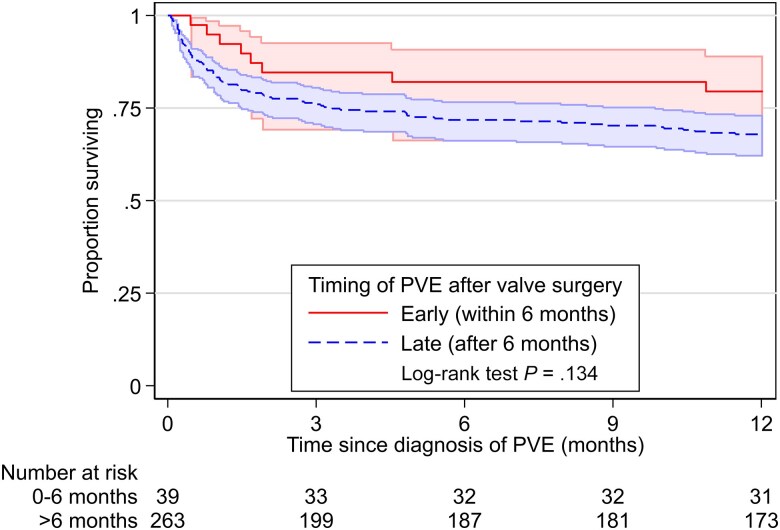
Kaplan-Meier analysis comparing mortality rates between early (≤6 months postsurgery [21%]) and late (>6 months postsurgery [32%]) prosthetic valve endocarditis (PVE) (log-rank test *P* = .134).

During 226 patient-years of follow-up, 92 patients died, resulting in 1-year mortality of 31% (95% confidence interval [CI]: 26%–36%), which was comparable between the 2 cohorts (34% vs 27%, log-rank test *P* = .193) (Supplementary Table 1). One-year mortality for early and late PVE was 21% (11%–37%) and 32% (27%–38%), respectively (*P* = .126) (Figure 1). Regarding surgical indications, 1-year mortality was 67% for heart failure, 40% for uncontrolled infection, and 30% for prevention of embolism. For patients with and without redo valve surgery, 1-year mortality was 23% (16%–32%) and 35% (28%–42%), respectively (*P* = .028). The final multivariable Cox model showed that CCI >4 points (adjusted hazard ratio [aHR], 2.06 [95% CI: 1.30–3.25]), presence of sepsis (aHR, 3.57 [95% CI: 2.25–5.67]), and *S. aureus* infection (aHR, 1.65 [95% CI: 1.09–2.49]) were independently associated with 1-year mortality, while redo cardiac surgery (aHR, 0.57 [95% CI: .34–.96]) was protective (Supplementary Table 2). Similar to the Kaplan–Meier failure curves, there was no evidence indicating an impact of timing of PVE on 1-year mortality in multivariable Cox models (aHR for PVE <6 months: 0.61 [95% CI: .28–1.32]; *P* = .21). Results from the sensitivity analysis without subsequent PVE episodes were similar (aHR, 0.65 [95% CI:.30–1.39]; *P* = .26).

Supplementary Table 3 presents a comparison between episodes of early and late PVE. We found no significant differences between early and late PVE across various indications for valve surgery (heart failure: 8% vs 10%, *P* = .779; uncontrolled infection: 62% vs 60%, *P* = .863; and prevention of embolism: 13% vs 19%, *P* = .503) or redo valve surgery while on antimicrobial treatment (44% vs 37%, *P* = .480). There was no difference in PVE recurrence between early and late PVE (8% vs 5%, *P* = .428).

## DISCUSSION

Our analysis of 302 PVE episodes revealed a 31% 1-year mortality. Notably, we found no evidence suggesting that early PVE leads to a worse outcome compared to late PVE.

Our results question the relevance of the new 2023 ESC guidelines recommendations, which advocate for surgical intervention in cases of early PVE [8]. The guidelines introduced this new criterion assuming that early PVE correlates with increased mortality and that relying solely on antimicrobial therapy is unlikely to result in complete recovery. However, this novel recommendation is based on 2 studies with differing definitions of early PVE (12 months postsurgery in Castillo et al and 2 months postsurgery in Wang et al), which diverge from the current 2023 ESC guidelines (defining early PVE as occurring 6 months postsurgery) [1, 3]. Of note, Castillo et al concluded that there is insufficient evidence to substantiate the benefits of surgery in patients with early PVE [3]. In the present study, 1-year mortality did not vary between episodes of early and late PVE, despite similar rates of surgical indications and performance of redo valve surgery across both groups.

In our study, surgical treatment for PVE was associated with improved survival rates compared to nonsurgical treatment regardless of the timing of surgery. As previously shown, PVE patients accepted for surgery had better outcomes [10]. The impact of redo valve surgery on mortality was influenced by the exclusion of patients considered high-risk surgical candidates from such procedures [11].

Albeit not consistently reported across all literature, *S. aureus* emerged as the predominant pathogen among PVE episodes in our study [1, 7, 12]. *Staphylococcus aureus* was linked to worse outcomes, as it is more frequently associated with the presence of sepsis and other complications such as acute heart failure and other surgical indications, factors often correlated with increased mortality [1–6, 12]. Regarding microbiology, early PVE had more infections with enterococci and showed a trend toward more coagulase-negative staphylococci, with the latter being frequently associated with cases of PVE occurring within the first year postsurgery [3, 4].

Several limitations should be noted. This is a study with some patients enrolled retrospectively, and a modest sample size, which may not be sufficient to address the potential benefits of redo valve surgery in early PVE. Additionally, this study draws on data from 2 Swiss tertiary-care referral centers, which may limit its generalizability to other settings. Furthermore, 41 episodes involved transcatheter aortic valve implantation, which is typically reserved for older and more comorbid populations. Moreover, various factors influence the decision to perform surgery, such as patients’ wish, age, and comorbidities, which could influence the impact of valve surgery on outcome. Last, we cannot exclude survivor bias introduced by patients dying before having a chance to undergo redo surgery for PVE. However, cardiac surgery was performed after a median of 11 days (IQR, 6–19 days) following the diagnosis of PVE, whereas patients died after a median of 31 days (IQR, 12–92 days) after the diagnosis of PVE.

In conclusion, our study questions the 2023 ESC recommendation for redo valve surgery in all patients within 6 months post–valve surgery. Our data do not clearly rule out a major benefit of surgical intervention for early PVE. Instead, our study calls for a more nuanced decision-making in managing patients with PVE.

## Supplementary Material

ciae392_Supplementary_Data

## Supplementary Data

Supplementary materials are available at *Clinical Infectious Diseases* online. Consisting of data provided by the authors to benefit the reader, the posted materials are not copyedited and are the sole responsibility of the authors, so questions or comments should be addressed to the corresponding author.

## Notes

***Author contributions.*** B. H. and M. P.-O. conceived the idea. M. P.-O., P. M., M. F., G. T., P. T., M. K., M. v. H., O. D., J. E., B. G., and B. H. collected the patients’ data. B. H. and M. P.-O. supervised the project. B. L., M. P.-O. and B. S. performed the analysis. All authors interpreted the results. M. P.-O. wrote the first draft of the manuscript. All authors participated in revising the manuscript and reviewed and approved the final submitted version.

***Acknowledgments.*** The authors thank all involved physicians, study nurses, and, most importantly, participants of the respective centers. The authors also thank Professor R. D. Kouyos for statistical advice.

***Disclaimer***. The funders had no role in study design, data collection and analysis, decision to publish, or preparation of the manuscript.

***Financial support***. This work was supported by the Swiss National Science Foundation (grant number 32003B_219351/1 to B. H. and M. P.-O.) and the Clinical Research Priority Program (CRPP) of the University of Zurich for the CRPP Precision medicine for bacterial infections (to B. H.).

## References

[1] Wang A, Athan E, Pappas PA, et al Contemporary clinical profile and outcome of prosthetic valve endocarditis. JAMA 2007; 297:1354–61. 17392239 10.1001/jama.297.12.1354

[2] Habib G, Erba PA, Iung B, et al Clinical presentation, aetiology and outcome of infective endocarditis. Results of the ESC-EORP EURO-ENDO (European Infective Endocarditis) registry: a prospective cohort study. Eur Heart J 2019; 40:3222–32. 31504413 10.1093/eurheartj/ehz620

[3] Castillo JC, Anguita MP, Torres F, et al Long-term prognosis of early and late prosthetic valve endocarditis. Am J Cardiol 2004; 93:1185–7. 15110221 10.1016/j.amjcard.2004.01.056

[4] Lopez J, Revilla A, Vilacosta I, et al Definition, clinical profile, microbiological spectrum, and prognostic factors of early-onset prosthetic valve endocarditis. Eur Heart J 2007; 28:760–5. 17255216 10.1093/eurheartj/ehl486

[5] Chirouze C, Alla F, Fowler VG, Jr, et al Impact of early valve surgery on outcome of *Staphylococcus aureus* prosthetic valve infective endocarditis: analysis in the International Collaboration of Endocarditis–Prospective Cohort Study. Clin Infect Dis 2015; 60:741–9. 25389255 10.1093/cid/ciu871 PMC4366581

[6] Ambrosioni J, Hernandez-Meneses M, Durante-Mangoni E, et al Epidemiological changes and improvement in outcomes of infective endocarditis in Europe in the twenty-first century: an International Collaboration on Endocarditis (ICE) prospective cohort study (2000–2012). Infect Dis Ther 2023;12:1083–101. 36922460 10.1007/s40121-023-00763-8 PMC10147876

[7] Fauchier L, Pericart L, Bourguignon T, et al Comparison of outcome of possible versus definite infective endocarditis involving prosthetic or bioprosthetic heart valves. Am J Cardiol 2017; 120:1884–90. 28917497 10.1016/j.amjcard.2017.07.100

[8] Delgado V, Ajmone Marsan N, de Waha S, et al 2023 ESC guidelines for the management of endocarditis. Eur Heart J 2023; 44:3948–4042. 37622656 10.1093/eurheartj/ehad193

[9] Habib G, Lancellotti P, Antunes MJ, et al 2015 ESC guidelines for the management of infective endocarditis: The Task Force for the Management of Infective Endocarditis of the European Society of Cardiology (ESC). Endorsed by: European Association for Cardio-Thoracic Surgery (EACTS), the European Association of Nuclear Medicine (EANM). Eur Heart J 2015; 36:3075–128. 26320109 10.1093/eurheartj/ehv319

[10] Shrestha NK, Shah SY, Hussain ST, et al Association of surgical treatment with survival in patients with prosthetic valve endocarditis. Ann Thorac Surg 2020; 109:1834–43. 31606518 10.1016/j.athoracsur.2019.09.015

[11] Van Hemelrijck M, Sromicki J, Frank M, et al Dismal prognosis of patients with operative indication without surgical intervention in active left-sided infective endocarditis. Front Cardiovasc Med 2023; 10:1223878. 37692048 10.3389/fcvm.2023.1223878 PMC10491846

[12] Mistiaen WP . What are the main predictors of in-hospital mortality in patients with infective endocarditis: a review. Scand Cardiovasc J 2018; 52:58–68. 29382232 10.1080/14017431.2018.1433318

