# Supplementary material for: Beyond the Timeline: 1-Year Mortality Trends in Early Versus Late Prosthetic Valve Endocarditis
Source: Clin Infect Dis. 2024 Jul 27;80(4):804–6. doi: 10.1093/cid/ciae392 (PMC12043058; doi:10.1093/cid/ciae392)
Supplement: ciae392_Supplementary_Data [file ciae392_supplementary_data.pdf]

Supplementary Table 1. Characteristics of 302 episodes with prosthetic valve endocarditis based on one-year survival

	Total (n=302)	Lost to follow up (n=6)	Survivors (n=206)	Deaths (n=92)	*P*[a]
**Demographics**					
Male sex, n (%)	240 (80)	4 (67)	160 (78)	76 (83)	0.408
Age (years), median (IQR)	68 (49-76)	59 (60-67)	66 (46-75)	72 (62-79)	0.001
Age >60 years, n (%)	197 (65)	3 (50)	121 (59)	73 (79)	0.001
CCI (points), median (IQR)	4 (2-6)	4 (1-6)	4 (1-6)	5 (3-7)	<0.001
CCI >4 points, n (%)	140 (46)	3 (50)	77 (38)	60 (65)	<0.001
Second episode, n (%)	16 (5)	0 (0)	10 (5)	6 (7)	0.568
**Cohort**					
CHUV, n (%)	170 (56)	1 (17)	112 (55)	57 (62)	0.256
USZ, n (%)	132 (44)	5 (83)	92 (45)	35 (38)	
**Microbiology**					
Staphylococcus aureus, n (%)	106 (35)	1 (17)	59 (29)	46 (50)	<0.001
Coagulase negative staphylococci, n (%)	18 (6)	0 (0)	13 (6)	5 (5)	1.000
Streptococcus spp, n (%)	70 (24)	0 (0)	54 (27)	16 (17)	0.089
Enterococcus spp, n (%)	43 (15)	0 (0)	35 (17)	8 (9)	0.056
Other Gram-positive, n (%)	15 (5)	0 (0)	13 (6)	2 (2)	0.159
HACEK, n (%)	14 (5)	1 (17)	10 (5)	3 (3)	0.761
Other Gram-negative, n (%)	8 (3)	2 (3)	6 (3)	0 (0)	0.182

Intracellular pathogens, n (%)	5 (2)	0 (0)	5 (3)	0 (0)	0.329
Fungi, n (%)	4 (1)	0 (0)	1 (0.5)	3 (3)	0.091
Polymicrobial infection, n (%)	8 (3)	0 (0)	7 (3)	1 (1)	0.442
Culture-negative, n (%)	27 (9)	2 (33)	15 (7)	10 (11)	0.314
**Clinical manifestations**					
Fever, n (%)	248 (82)	6 (100)	166 (81)	746 (83)	0.799
Acute heart failure, n (%)	79 (26)	0 (0)	38 (19)	41 (45)	<0.001
Embolic events, n (%)	147 (49)	1 (17)	95 (47)	51 (55)	0.158
Cerebral embolic events, n (%)	103 (34)	1 (17)	65 (32)	37 (40)	0.162
Non-cerebral embolic events, n (%)	98 (33)	1 (17)	63 (31)	34 (37)	0.303
Immunologic phenomena, n (%)	12 (4)	0 (0)	8 (4)	4 (4)	1.000
Sepsis, n (%)	118 (39)	0 (0)	59 (29)	59 (64)	<0.001
**Site of infection**					
Aortic valve, n (%)	208 (69)	4 (67)	139 (68)	65 (71)	0.665
Mitral valve, n (%)	90 (30)	2 (33)	55 (27)	33 (36)	0.121
Tricuspid valve, n (%)	14 (5)	1 (17)	9 (4)	4 (3)	1.000
Pulmonary valve, n (%)	23 (8)	1 (17)	21 (10)	1 (1)	0.003
Several valves affected, n (%)	34 (11)	2 (33)	20 (10)	12 (13)	0.442
**Type of prosthetic valve**					
Biological valve, n (%)	184 (61)	6 (100)	121 (59)	57 (62)	0.671[b]
Mechanical valve, n (%)	77 (26)	0 (0)	56 (28)	21 (23)	

TAVI, n (%)	41 (14)	0 (0)	27 (13)	14 (15)	
**Type of intracardiac lesions**					
Any vegetation, n (%)	215 (71)	5 (83)	146 (72)	64 (70)	0.725
Vegetation ≥10mm, n (%)	62 (21)	0 (0)	48 (24)	14 (15)	0.104
Abscess, n (%)	52 (18)	0 (0)	35 (17)	17 (19)	0.782
Dehiscence of prosthetic valve, n (%)	37 (13)	0 (0)	27 (13)	10 (11)	0.569
Fistula, aneurysm, or pseudoaneurysm, n (%)	20 (7)	0 (0)	10 (5)	10 (11)	0.058
New valvular regurgitation, n (%)	22 (7)	0 (0)	16 (8)	6 (7)	0.813
Abnormal metabolic activity in ^{18}F-FDG PET/CT, n (%)	72 (24)	3 (100)	50 (25)	19 (21)	0.468
Concomitant CIED-lead, n (%)	22 (7)	1 (17)	13 (6)	8 (9)	0.471
**Classification according to 2023 Duke-ISCVID**					
Definite, n (%)	251 (83)	4 (67)	169 (83)	78 (85)	0.678
Possible, n (%)	51 (17)	2 (33)	35 (17)	14 (15)	
**Indications for valve surgery**, n (%)	203 (67)	3 (50)	124 (61)	76 (83)	<0.001
Heart failure, n (%)	30 (10)	0 (0)	10 (5)	20 (22)	<0.001
Uncontrolled infection, n (%)	181 (60)	3 (50)	107 (53)	71 (77)	<0.001
Prevention of embolism, n (%)	54 (18)	0 (0)	38 (19)	16 (17)	0.872
**Timing of PVE after valve surgery**					
Early (within 6 months), n (%)	39 (13)	0 (0)	31 (15)	8 (9)	0.126
Late (after 6 months), n (%)	263 (87)	6 (100)	173 (85)	84 (91)	
**Redo valve surgery during antimicrobial treatment**, n (%)	114 (38)	2 (33)	86 (42)	26 (28)	0.028

[a] comparison between survivors and non-survivors

[b]comparison between TAVI and both mechanical and biological prosthetic valves

^{18}F-FDG PET/CT: ^{18}F-Fluorodeoxyglucose Positron Emission Tomography/Computed Tomography; CCI: Charlson Comorbidity Index; CHUV: Lausanne University Hospital; CIED: cardiac implantable electronic devices; HACEK: *Haemophilus* spp, *Aggregatibacter* spp, *Cardiobacterium hominis*, *Eikenella corrodens*, *Kingella kingae*; ISCVID: International Society of Cardiovascular Infectious Diseases; IQR: interquartile range; PVE: prosthetic valve endocarditis; TAVI: Transcatheter aortic valve implantation; USZ: University Hospital Zurich

Supplementary Table 2. Univariable analysis and multivariable Cox proportional hazards regression models of one-year mortality among patients with prosthetic valve endocarditis

	Univariable analysis		Multivariable analysis	
	P	**HR (95% CI)**	***P***	**aHR (95% CI)**
Early PVE (within 6 months)	0.103	0.55 (0.27-1.12)	0.210	0.61 (0.28-1.32)
Male sex	0.345	1.29 (0.76-2.23)	0.103	1.49 (0.92-2.41)
CCI >4 points	<0.001	2.33 (1.52-3.58)	0.002	2.06 (1.30-3.26)
S. aureus	<0.001	2.25 (1.50-3.37)	0.019	1.65 (1.09-2.49)
Sepsis	<0.001	3.76 (2.43-5.81)	<0.001	3.57 (2.25-5.67)
Redo valve surgery during antimicrobial treatment	0.030	0.61 (0.38-0.95)	0.033	0.57 (0.34-0.96)

aHR: adjusted hazard ratio; CCI: Charlson Comorbidity Index; CI: confidence interval; PVE: prosthetic valve endocarditis

Supplementary Table 3. Characteristics of 302 episodes based on the timing of prosthetic valve endocarditis occurrence after valve surgery

	Early PVE (within 6 months) (n=39)	Late PVE (after 6 months) (n=263)	***P***
**Demographics**			
Male sex, n (%)	32 (82)	208 (79)	0.832
Age (years), median (IQR)	73 (63-79)	67 (48-76)	0.008
Age >60 years, n (%)	32 (82)	165 (63)	0.019
CCI (points), median (IQR)	5 (3-7)	4 (2-6)	0.137
CCI >4 points, n (%)	21 (53)	119 (45)	0.390
Second episode, n (%)	0 (0)	16 (6)	0.240
**Cohort**			
CHUV, n (%)	26 (67)	144 (55)	0.171
USZ, n (%)	13 (33)	119 (45)	
**Microbiology**			
Staphylococcus aureus, n (%)	13 (33)	93 (35)	0.859
Coagulase negative staphylococci, n (%)	5 (13)	13 (5)	0.066
Streptococcus spp, n (%)	9 (23)	61 (23)	1.000
Enterococcus spp, n (%)	10 (26)	33 (13)	0.046
Other Gram-positive, n (%)	1 (3)	14 (5)	0.702
HACEK, n (%)	0 (0)	14 (5)	0.229
Other Gram-negative, n (%)	0 (0)	8 (3)	0.602

Intracellular pathogens, n (%)	0 (0)	5 (2)	1.000
Fungi, n (%)	0 (0)	4 (2)	1.000
Polymicrobial infection, n (%)	3 (8)	5 (2)	0.070
Culture-negative, n (%)	4 (10)	23 (9)	0.763
**Cardiac imaging studies**			
TTE, n (%)	37 (95)	249 (95)	1.000
TEE and/or ^{18}F-FDG PET/CT or cardiac CT, n (%)	33 (85)	228 (87)	0.802
TEE, n (%)	29 (74)	205 (78)	0.681
^{18}F-FDG PET/CT or cardiac CT, n (%)	12 (31)	95 (36)	0.593
**Clinical manifestations**			
Fever, n (%)	30 (77)	218 (83)	0.373
Acute heart failure, n (%)	9 (23)	70 (27)	0.701
Embolic events, n (%)	16 (41)	131 (50)	0.391
Cerebral embolic events, n (%)	11 (28)	92 (35)	0.472
Non-cerebral embolic events, n (%)	9 (23)	89 (34)	0.204
Immunologic phenomena, n (%)	1 (3)	11 (4)	1.000
Sepsis, n (%)	12 (31)	106 (40)	0.294
**Site of infection**			
Aortic valve, n (%)	27 (69)	181 (69)	1.000
Mitral valve, n (%)	14 (36)	76 (29)	0.453
Tricuspid valve, n (%)	3 (8)	11 (4)	0.403

Pulmonary valve, n (%)	0 (0)	23 (9)	0.054
Several valves affected, n (%)	6 (15)	28 (11)	0.414
**Type of prosthetic valve**			
Biological valve, n (%)	23 (59)	161 (60)	0.002[a]
Mechanical valve, n (%)	4 (10)	73 (28)	
TAVI, n (%)	12 (31)	29 (11)	
**Type of intracardiac lesions**			
Vegetation, n (%)	27 (69)	188 (72)	0.850
Vegetation ≥10mm, n (%)	6 (15)	56 (21)	0525
Abscess, n (%)	11 (28)	52 (16)	0.067
Dehiscence of prosthetic valve, n (%)	6 (15)	31 (12)	0.599
Fistula, aneurysm, or pseudoaneurysm, n (%)	5 (13)	15 (6)	0.156
New valvular regurgitation, n (%)	3 (8)	19 (7)	1.000
Abnormal metabolic activity in ^{18}F-FDG PET/CT, n (%)	9 (23)	63 (24)	1.000
Concomitant CIED-lead , n (%)	5 (13)	17 (7)	0.180
**Classification according to 2023 Duke-ISCVID**			
Definite, n (%)	30 (77)	221 (84)	0.259
Possible, n (%)	9 (23)	42 (16)	
**Indications for valve surgery**, n (%)	26 (67)	177 (67)	1.000
Heart failure, n (%)	3 (8)	27 (10)	0.779
Uncontrolled infection, n (%)	24 (62)	157 (60)	0.863

Prevention of embolism, n (%)	5 (13)	49 (19)	0.503
**Redo valve surgery during antimicrobial treatment**, n (%)	17 (44)	97 (37)	0.480
**Lost to follow up**, n (%)	0 (0)	6 (2)	1.000
**One-year mortality or recurrence**, n (%)[b]	10 (26)	92 (36)	0.214
Mortality, n (%)[b]	8 (21)	84 (33)	0.126
Recurrence, n (%)[b]	3 (8)	12 (5)	0.428

[a]comparison between TAVI and both mechanical and biological prosthetic valves

[b]among 296 episodes without lost to follow up

^{18}F-FDG PET/CT: ^{18}F-Fluorodeoxyglucose Positron Emission Tomography/Computed Tomography; CCI: Charlson Comorbidity Index; CHUV: Lausanne University Hospital; CIED: cardiac implantable electronic devices; HACEK: *Haemophilus* spp, *Aggregatibacter* spp, *Cardiobacterium hominis*, *Eikenella corrodens*, *Kingella kingae*; ISCVID: International Society of Cardiovascular Infectious Diseases; IQR: interquartile range, TAVI: Transcatheter aortic valve implantation; TEE: transesophageal echocardiography; TTE: transthoracic echocardiography; USZ: University Hospital Zurich
